# Six-year diagnostic delay in a married woman with Mayer–Rokitansky–Küster–Hauser syndrome: a case report

**DOI:** 10.1186/s13256-026-06494-x

**Published:** 2026-08-13

**Authors:** Sarah K. Kebbeh, Safinaz J. Dahoud, Salam Arab, Mohammad Feras Dhemish

**Affiliations:** 1 University of Aleppo, Aleppo, Syria; 2 Department of Obstetrics and Gynecology, Dar Al Tawleed State Hospital, Aleppo, Syria

**Keywords:** Mayer–Rokitansky–Küster–Hauser syndrome (MRKH), Primary amenorrhea, Uterine agenesis, Müllerian Ducts anomalies, Case report

## Abstract

**Background:**

Mayer–Rokitansky–Küster–Hauser (MRKH) syndrome type 1 is typically diagnosed during adolescence, with primary amenorrhea being the most common presenting symptom in young women with otherwise normal secondary sexual characteristics and external genitalia. Patients with MRKH syndrome lack a uterus and the upper two-thirds of the vagina. The syndrome has a prevalence of approximately 1 in 4,500 live female births, making it a rare condition.

**Case presentation:**

We report the case of a 27-year-old, Syrian, Arab married woman who presented with primary amenorrhea and a desire to conceive. Despite multiple clinical evaluations over several years, she remained undiagnosed until her presentation to our clinic. The diagnosis of MRKH syndrome was confirmed through pelvic ultrasound, magnetic resonance imaging (MRI), and diagnostic laparoscopy.

**Conclusions:**

This case is notable not only for the rarity of MRKH syndrome, but also for the 6-year delay in diagnosis despite ongoing symptoms. It underscores the critical need for early and thorough gynecologic evaluation in adolescents and young women presenting with primary amenorrhea, even in the presence of normal secondary sexual characteristics. The case also highlights how cultural and religious factors may influence reproductive decision-making and limit available fertility options in patients with MRKH.

**Supplementary Information:**

The online version contains supplementary material available at https://doi.org/10.1186/s13256-026-06494-x.

## Introduction

Mayer–Rokitansky–Küster–Hauser (MRKH) syndrome, or Müllerian aplasia, is a congenital condition characterized by the absence or underdevelopment of the uterus and upper two-thirds of the vagina in individuals with normal secondary sexual characteristics and a 46,XX karyotype [1].

The syndrome affects approximately 1 in 4,500 female live births, though this figure remains imprecise due to limited epidemiological data [1, 2]. MRKH results from the failed development or fusion of the paramesonephric (Müllerian) ducts during embryogenesis, typically around the 5th to 6th gestational week [1].

Patients usually present in adolescence with primary amenorrhea, despite normal pubertal development. Other symptoms may include dyspareunia or cyclic pelvic pain. The median age at diagnosis is around 17.5 years [1].

Initial assessment often involves pelvic ultrasound, which may reveal absent uterus and normal ovaries [1]. MRI is the diagnostic gold standard for confirming uterovaginal agenesis. Renal anomalies occur in about 30% of cases and should be screened with imaging [1].

First-line treatment focuses on vaginal dilation therapy for neovagina creation [3].

Uterus transplantation is an emerging fertility option [3].

In many conservative and resource-limited settings, discussion of menstrual and reproductive health remains limited, which may contribute to delayed recognition and evaluation of primary amenorrhea. Cultural sensitivity, social stigma, and lack of sexual health education can prevent young women from seeking timely medical attention, even when clear symptoms, such as absence of menarche are present.

This delay may lead to inappropriate or prolonged empirical treatments without adequate anatomical evaluation. Early and structured assessment of primary amenorrhea, including pelvic imaging, is essential to avoid missed or delayed diagnoses, particularly in conditions, such as MRKH syndrome, where secondary sexual characteristics are normal [4, 5].

We present a rare case of a 27-year-old woman with MRKH syndrome diagnosed 6 years after initial presentation, highlighting the impact of delayed evaluation, the role of socio-cultural factors, and the importance of early and comprehensive assessment of primary amenorrhea.

## Case presentation

A 27-year-old, Syrian, Arab woman, married for 6 years, presented to our gynecology clinic with a desire to conceive. She reported primary amenorrhea, with no history of menarche. There were no external genital abnormalities, no similar familial history, and her mother denied any medication use during pregnancy.

The patient had completed education up to the ninth grade and resided in a rural area. She reported only limited and traditional knowledge regarding menstrual and reproductive health prior to marriage, with no formal sexual education. There was no family history of primary amenorrhea, delayed puberty, or known Mullerian anomalies among her first-degree relatives or siblings.

Since 2019, the patient had been evaluated by several clinicians. She had been prescribed combined estrogen–progestin therapy empirically to induce withdrawal bleeding, with bimonthly ultrasound follow-up, but menstruation did not occur. She described her marital relationship as normal, including satisfactory sexual intercourse.

On examination, the patient had a normal female phenotype, with well-developed breasts corresponding to Tanner stage V. External genitalia were normal.

A pelvic ultrasound, performed at our clinic, revealed a small, hypoplastic uterus measuring approximately 16 × 18 × 20 mm, with bilaterally normal-appearing ovaries. No adnexal masses or cysts were identified.

An attempt at hysterosalpingography (HSG), performed at an outside facility prior to referral, was unsuccessful due to the absence of an identifiable cervical canal. A blind vaginal pouch was noted on clinical examination, suggesting vaginal aplasia.

Previous laboratory investigations showed:

- TSH: 0.9 mIU/L
- FT4: 16 pmol/L
- Prolactin: 18 ng/mL
- FSH: 2.4 IU/L
- LH: 6.3 IU/L
- 17-OH progesterone: 1.7 ng/mL
- Free testosterone: 0.4 ng/mL
- Estradiol: 121 pmol/L.

All values were within normal limits.

A previous brain and pituitary MRI (performed at another center) revealed normal findings.

At our center, pelvic MRI (T1 and T2 sequences, with and without contrast) revealed a narrow, hypoplastic vagina approximately 5 cm in length, terminating in a fibrotic rudimentary uterine bud. The ovaries appeared normal in size and signal. No pelvic masses or cystic lesions were identified. The urinary bladder, rectum, and sigmoid colon were unremarkable. Both kidneys were present and appeared normal. No vertebral or skeletal abnormalities were identified on imaging. These findings supported the diagnosis of isolated MRKH syndrome (Type 1).

A diagnostic laparoscopy confirmed complete uterine agenesis with normal bilateral adnexa, consistent with a congenital Müllerian duct anomaly (Table 1; Video 1).

**Table 1:** Diagnostic workup sequence

Step	Investigations	Findings
1	Hormonal profile	Normal FSH, LH, estradiol
2	Pelvic ultrasound	Hypoplastic uterus, normal ovaries
3	Hysterosalpingography	Failed (no cervical canal)
4	MRI pelvis	Uterovaginal agenesis
5	Diagnostic laparoscopy	Confirmed MRKH syndrome

Based on the clinical history, imaging, and laparoscopic findings, the patient was diagnosed with Mayer–Rokitansky–Küster–Hauser (MRKH) syndrome.

Following diagnosis disclosure, the patient experienced emotional distress and was offered psychological counseling, which she declined due to cultural stigma associated with mental health services.

She expressed a strong desire for biological motherhood and declined gestational surrogacy for personal religious reasons. She was counseled regarding available reproductive options and non-surgical vaginal dilation as a conservative treatment with planned follow-up.

This case reflects a delayed diagnosis, likely due to initial misinterpretation and prolonged empirical hormonal therapy without early anatomical assessment.

This timeline summarizes the delayed diagnostic pathway and multiple prior evaluations before definitive diagnosis (Table 2).

**Table 2:** Clinical timeline of the patient

Time/Age	Clinical event
21 years	Marriage
2019	First medical evaluation for primary amenorrhea
2019–2025	Repeated consultations with empirical hormonal therapy and follow-up ultrasounds without menstruation
During this period	Hysterosalpingography attempted but failed due to absent cervical canal
2025	Presentation to our clinic for infertility assessment
2025	Pelvic ultrasound, MRI, and diagnostic laparoscopy confirming MRKH syndrome
Post-diagnosis	Counseling and explanation of fertility options and management plan

## Discussion

Mayer–Rokitansky–Küster–Hauser (MRKH) syndrome affects approximately 1 in 4,500 live female births [2]. It is classified into type I (isolated uterovaginal agenesis) and type II (associated with renal, skeletal, or other anomalies) [2]. This case involves type I MRKH, though the incidence of this subtype varies across studies and remains imprecise [2].

The exact cause of MRKH is still unclear. While initially believed to be sporadic and potentially linked to environmental factors, no such associations have been confirmed [1]. Evidence now supports a genetic basis, with mutations in genes such as WNT4, LHX1, and HNF1B reported in some cases [1]. In this case, the patient’s relatives had no signs of Müllerian anomalies and were fertile. She reported no in-utero drug exposure, and her development appeared typical.

The patient experienced primary amenorrhea but delayed medical evaluation until after marriage. This delay reflects a need for greater awareness and earlier investigation of delayed menarche in adolescents. She received empirical hormonal therapy (combined estrogen–progestin therapy) for 10 months under the assumption of a functional disorder. Hormonal tests, including FSH, LH, and estradiol, were normal, and ultrasounds showed no uterine response. A pituitary MRI was performed to exclude central causes. These steps illustrate a common diagnostic oversight: delaying pelvic imaging in cases, where hormonal profiles are normal and secondary sexual characteristics are present [4].

This case also highlights the importance of early pelvic imaging in patients with primary amenorrhea and normal secondary sexual characteristics. Relying solely on hormonal evaluation may delay accurate diagnosis of structural Müllerian anomalies, such as MRKH syndrome.

Delayed diagnosis of MRKH syndrome, although uncommon, may have significant psychological, social, and reproductive implications. Patients may undergo unnecessary investigations or empirical hormonal treatments before proper anatomical evaluation is performed. Previous studies have shown that MRKH syndrome may negatively affect self-image, sexuality, psychological well-being, and quality of life, emphasizing the importance of timely diagnosis and psychological support [6].

In this case, delayed diagnosis may be attributed to multiple factors, including limited awareness of primary amenorrhea as a pathological condition, delayed presentation after marriage, and reliance on empirical hormonal therapy without early imaging assessment. In addition, socio-cultural barriers and lack of structured sexual and reproductive health education may contribute to postponing medical consultation.

Management of MRKH syndrome requires a multidisciplinary approach, including gynecological care, psychological support, and counseling regarding fertility options. According to current clinical guidelines, early diagnosis and appropriate counseling are essential to minimize psychological distress and improve quality of life [4, 5].

Gynecological care of MRKH focuses on creating a functional vagina to enable sexual activity. Non-surgical dilation is the first-line approach; surgery is reserved for unsuccessful cases [5].

Although patients lack a uterus, IVF with a gestational carrier may allow biological parenthood when ovarian function is intact. In this case, religious beliefs prohibit the use of gestational surrogacy, limiting reproductive options and highlighting the need for culturally sensitive counseling. Psychological support is also essential to help patients cope with the emotional and social impact of the diagnosis [5]. Recent systematic reviews have demonstrated higher rates of anxiety, depression, social insecurity, and impairment in mental health-related quality of life among women with MRKH syndrome, further supporting the need for multidisciplinary care and long-term psychosocial support [7].

## Conclusion

This case emphasizes that primary amenorrhea should always prompt timely and systematic evaluation, including pelvic imaging, regardless of normal secondary sexual characteristics. Increased awareness and sexual health education are crucial to prevent delayed diagnosis in similar cases.

Early recognition, culturally appropriate counseling, and multidisciplinary management are key to optimizing outcomes in these patients.

Cultural and religious factors also influenced reproductive decision-making, highlighting the importance of culturally sensitive counseling.

## Supplementary Information

Supplementary material 1. Recorded laparoscopic views complete uterine agenesis with normal bilateral adnexa

## Data Availability

Not applicable.
